# Recapitulating X-Linked Juvenile Retinoschisis in Mouse Model by Knock-In Patient-Specific Novel Mutation

**DOI:** 10.3389/fnmol.2017.00453

**Published:** 2018-01-12

**Authors:** Ding Chen, Tao Xu, Mengjun Tu, Jinlin Xu, Chenchen Zhou, Lulu Cheng, Ruqing Yang, Tanchu Yang, Weiwei Zheng, Xiubin He, Ruzhi Deng, Xianglian Ge, Jin Li, Zongming Song, Junzhao Zhao, Feng Gu

**Affiliations:** ^1^State Key Laboratory of Ophthalmology, Optometry and Vision Science, School of Ophthalmology and Optometry, Eye Hospital, Wenzhou Medical University, Wenzhou, China; ^2^Ministry of Health and Zhejiang Provincial Key Laboratory of Ophthalmology and Optometry, Wenzhou, China; ^3^The Second Affiliated Hospital and Yuying Children’s Hospital of Wenzhou Medical University, Wenzhou, China; ^4^Department of Ophthalmology, Henan Provincial People’s Hospital, People’s Hospital of Zhengzhou University, Zhengzhou, China

**Keywords:** retina, X-linked juvenile retinoschisis, knock-in, mice, disease model, genome editing technologies

## Abstract

X-linked juvenile retinoschisis (XLRS) is a retinal disease caused by mutations in the gene encoding retinoschisin (RS1), which leads to a significant proportion of visual impairment and blindness. To develop personalized genome editing based gene therapy, knock-in animal disease models that have the exact mutation identified in the patients is extremely crucial, and that the way which genome editing in knock-in animals could be easily transferred to the patients. Here we recruited a family diagnosed with XLRS and identified the causative mutation (*RS1*, p.Y65X), then a knock-in mouse model harboring this disease-causative mutation was generated via TALEN (transcription activator-like effector nucleases). We found that the b-wave amplitude of the ERG of the *RS1*-KI mice was significantly decreased. Moreover, we observed that the structure of retina in *RS1*-KI mice has become disordered, including the disarray of inner nuclear layer and outer nuclear layer, chaos of outer plexiform layer, decreased inner segments of photoreceptor and the loss of outer segments. The novel knock-in mice (*RS1*-KI) harboring patient-specific mutation will be valuable for development of treatment via genome editing mediated gene correction.

## Introduction

X-linked juvenile retinoschisis (XLRS) is a common X-linked recessive genetic disease of macular degeneration which typically begins in young boys and leads to blindness throughout life. There are, as yet, no effective treatments for this disease in the clinic. It was first characterized in two brothers in Germany in 1898 by Haas (Molday et al., 2012), with an estimated prevalence of 1/5,000 to 1/25,000 (Sauer et al., 1997). Typical electroretinograms (ERG) of retinoschisis reveals reduced b-wave amplitude with relative preservation of the a-wave. The hallmark of XLRS is the presence of a spoke-wheel pattern in the macula in patients. These cavities are readily observed via optical coherence tomography (OCT).

X-linked juvenile retinoschisis is caused by mutations in the Retinoschisin 1 (*RS1*) gene located on chromosome Xp22.2 It is composed of six exons and encoded 224-amino acid protein known as retinoschisin. The 24-kDa retinoschisin is a cell adhesion protein, which is composed of a *RS1* domain and a single discoidin domain containing protein (Sauer et al., 1997; Wu and Molday, 2003). This protein binds tightly to the surface of photoreceptors and bipolar cells where it helps to maintain the cellular organization of the retina and structure of the photoreceptor-bipolar synapse (Molday et al., 2007). To date, more than 210 mutations have been identified (Vincent et al., 2013), such as missense mutations, deletions, insertions, splice site mutations and so on^1^.

To develop clinical treatments, animal disease model is crucial. So far, three *RS1*-KO mouse models have been developed. Disruption of the *RS1* gene was accomplished by targeting exon 3 with an in-frame *lacZ-neo^r^* expression cassette (Weber et al., 2002). The second model was generated by replacing exon 1 to intron 1 of the murine *RS1* gene with a neomycin resistance (*neo^r^*) gene cassette (Zeng et al., 2004). *44TNJ* mouse disease model, as the third one, was generated by *N*-ethyl-*N*-nitrosourea (ENU) mutagenesis (Jablonski et al., 2005), which leads to the introduction of novel splice variants. Based on these disease models, efforts are now being directed to develop a gene therapy approach for XLRS patients. Some studies investigated the potential of gene replacement therapy to restore retinal structure and function with knock-out mice as an XLRS animal disease model (Zeng et al., 2004, 2016; Dyka and Molday, 2007; Byrne et al., 2014; Marangoni et al., 2014, 2017; Apaolaza et al., 2015; Ou et al., 2015; Ye et al., 2015). Traditional gene replacement therapy does have gene silence and random integration issues. While, so far, as to the permanent effective treatment with *in situ* gene correction, it isn’t available.

Genome editing based gene therapy would be a great promising method for treating genetic diseases (Yin et al., 2014; Suzuki et al., 2016; Yang et al., 2016). However, disease models harboring the exact mutations identified in the patients are critical; because the treatment developed from these disease models with genome editing mediated *in situ* gene correction could be easily transferred to the patients. So far, knock-in mice with patient-specific *RS1* mutation have not been reported, which would be invaluable for development of novel therapies. To address it, we recruited a patient from a XLRS family and identified the causative mutation. Then knock-in mouse disease model harboring patient-specific mutation was generated by transcription activator-like effector nucleases (TALENs). This novel *RS1*-KI mouse will pave away for *in situ* gene correction based gene therapy and also be valuable for the studies of pathological mechanism for XLRS.

## Materials and Methods

### Patient Recruitment

This study conformed to the tenets of the Declaration of Helsinki. It was approved by the Ethics Committee of Eye Hospital, Wenzhou Medical University. Written informed consent was obtained from the recruited individuals. All experiments were performed in accordance with the approved guidelines. OCT, ERG and fundus examination were performed as routine retinal ophthalmic examination. A five ml venous blood sample was drawn into an ethylenediaminetetraacetic acid (EDTA) sample tube. Genomic DNA was extracted from peripheral blood leukocytes using the standard phenol/chloroform extraction protocols.

### Sequencing and Bioinformatics Analysis

Sequencing was performed as previously described (Zhang et al., 2014). No synonymous variants were evaluated by three algorithms, SIFT^2^, PolyPhen^3^ and PANTHER^4^ to determine pathogenicity. Multiple sequence alignments were performed using ESPript3.0^5^.

### Construction of TALEN Expression Vectors

Using the TALEN-Designer as described in Wefers et al. (2013), we constructed a TALEN pair against a target sequence within the fourth exon of *RS1* (TALEN *RS1*; **Figure 2A**). For each target sequence, we constructed TALEN coding regions using a pair of ligation reactions that each combines seven TAL repeat coding DNA segments, in the order specified by the variable 14–15 bp sequence that follows the invariable T at the first position.

### Oligodeoxynucleotides

The oligodeoxynucleotides ODN*^RS1^*(5′-TGGGTGTTTGAGTGTGTGCTGTTTTTCCTCCCCAGAATGCCC**t**TA**G**TCACAAGCCCCTGGGTTTCGAGTCAGGGGAGGTCACGCCAGATCA-3′) was synthesized and HPLC purified by Shanghai Sangon (Shanghai Sangon, China), each having a length of 91 nt, including the causative mutation (underlined) and a silent mutation (shown in boldface type and lower case), covering 45-bp upstream and downstream of the targeted site.

### *In Vitro* Transcription and Preparation of TALEN-Coding mRNA and Microinjection of One-Cell Embryos

TALEN mRNA was prepared using mMessage mMachine Sp6 Kit (Ambion, United States) following the manufacturer’s instructions. TALEN mRNA was co-injected into fertilized eggs with oligo donor for KI mice production. We followed the reported protocol (Wefers et al., 2013). Mice were handled according to institutional guidelines approved by the animal welfare and used committee of the Committee on the Ethics of Animal Experiments of the Wenzhou Medical University and housed in standard cages in a specific pathogen-free facility on a 12-h light/dark cycle with ad libitum access to food and water.

### PCR, Digestion, and Sequence Analysis

Genomic DNA was isolated from tail tips or toe clips of founder mice and their progenies, using the Wizard Genomic DNA Purification Kit (Promega, United States), and the sequence harboring the target site was amplified by PCR using the PCR primers: P-forward (P-for) (5′-GTGCCTATGTTTCTTGCTTTGT-3′) and P-reverse (P-rev) (5′-GAGGAATACCAGCCCACATAC-3′). Amplification was performed using Taq DNA Polymerase (Takara, Japan) in 25-μL reactions with 30 cycles of 95°C for 30 s, 59°C for 30 s, and 72°C for 30 s. The purified PCR products were digested with 10 units of Bpu10I and analyzed on agarose gels. DNA sequencing was used for confirmation of the genotyping results.

### RS1 Protein Immunohistochemistry

*RS1*-KI male mice and wild-type (WT) male mice were used in this study. Animals were anesthetized by intraperitoneal ketamine (100 mg/kg) and xylazine (10 mg/kg). The retinas from WT and *RS1*-KI mice were dissected, fixed overnight in 4% paraformaldehyde, and either cut into smaller pieces for whole-mount staining or processed for either paraffin section or frozen section by standard methods. The sections were stained using a rabbit polyclonal antibody against retinoschisin (Sigma, Germany) and a secondary antibody conjugated to green-fluorescent Alexa Fluor 488 dye (Beyotime, China). Nuclei were stained with DAPI.

### Western Blotting

Retinas from WT or *RS1*-KI mice were lysed in RIPA buffer with PMSF, pH 7.4 (Beyotime, China). Total protein was determined based on the bicinchoninic acid (BCA) method using the BCA Protein Assay Kit (Beyotime, China). Equal amounts of protein were separated by SDS-PAGE and transferred to PVDF membranes. The membranes were blocked with 5% milk dissolved in TBST (0.05 M Tris-buffered saline with 0.1% Tween-20) and then incubated overnight with the RS1-3R10 antibodies (gift from Dr. Laurie L. Molday). After overnight incubation, the membranes were washed with TBST and incubated with secondary antibody (IRDye 680RD-conjugated donkey anti-mouse, 1:2000). Finally, the membranes were washed with TBST three times for 5 min each time and the proteins were detected using the Odyssey two-color infrared laser imaging system. Protein levels were normalized to GAPDH (GAPDH polyclonal antibody, Bioworld, United States, 1:5000. Secondary antibody, IRDye 800CW conjugated goat anti-rabbit IgG, Odyssey, United States, 1:2000) signal.

### Statistical Analysis

Electroretinograms amplitude and OCT results were compared between WT and *RS1*-KI mice. The average raw score (a-wave amplitude, b-wave amplitude, b-/a-wave amplitude ratio, OS and IS thickness, ONL thickness) was calculated and the non-parametric one-way ANOVA with Mann–Whitney *U*-test was used to compare the difference. In all analysis, *P* < 0.05 was considered to be statistically significant.

## Results

### Clinical Data

**FIGURE 1:**
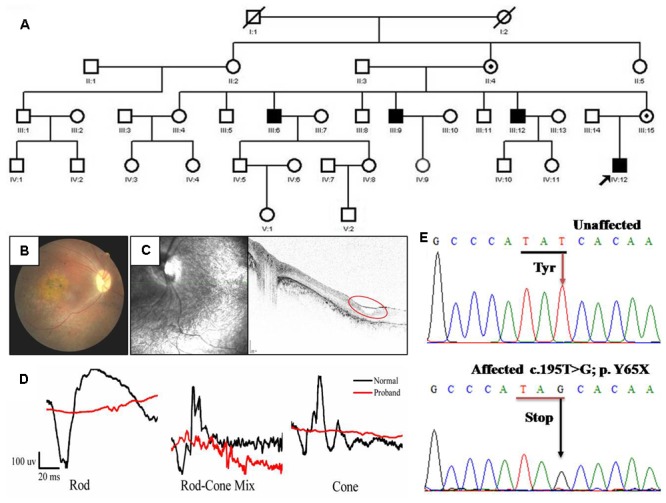
Pedigree and clinical characteristics of a patient with X-linked juvenile retinoschisis (XLRS). **(A)** Pedigree of the XLRS family. Black boxes, affected males; white boxes, unaffected males; white circles, unaffected females; boxes and circles with slashed, deceased; Circles with a black dot, carrier; the arrow points to the proband. **(B)** Fundoscopy of the proband patient revealed extensive retinal degeneration. Stellate maculopathy and retinal pigment epithelial atrophy in retinal fovea bilaterally. **(C)** Optical coherence tomography images from the proband, showing the retinal thickness is significantly decreased and cysts in peripheral retina (red circle). **(D)** The ERG of the rod, rod-cone mix, cone. Both a-wave and b-wave in XLRS proband are reduced or nearly disappeared. Black line, normal control; red line, proband. **(E)** Sanger sequencing results of unaffected individual and proband. T >G transversion in exon 4 (c.195T >G mutation) of *RS1* that causes a conservative substitution of tyrosine to a stop codon.

The family in our study is comprised of four effected individuals from a five-generation pedigree (**Figure 1A**). The proband is a 19-year-old male (IV: 12). The inheritance model of this family is X-linked as indicated by the pedigree. This proband showed a phenotype of stellate maculopathy and retinal pigment epithelial atrophy in retinal fovea bilaterally, with vascular attenuation or sheathing (**Figure 1B**), retinal pigment deposition, nystagmus, low vision (about 20/400), esotropia in the right eye (deviations 10°). His mother is asymptomatic with no clinical features.

The tiny cavities in the retina are readily seen by OCT as shown in **Figure 1C**. Inner and outer foveal thickness and volume were increased, as well as the outer retinal perifoveal and parafoveal thicknesses, with full-thickness and volume measurements decreased, and vertical palisades spanning the cleft between the inner nuclear layer and outer plexiform layer giving rise to the cystic-like spaces in the perifoveal region. Both the ERG a-wave and b-wave amplitude nearly disappeared (**Figure 1D**). Taken together, based on the clinical manifestations and pedigree, the diagnosis of the patient from this family is putative XRLS.

### Identification and Analysis of p.Y65X Mutation in *RS1*

To identify the genetic defects in this family, we performed DNA sequencing to screen the mutation. All exons and exon–intron boundaries sequences of the *RS1* gene were amplified by PCR and the products were analyzed by direct sequencing. We identified a novel nonsense mutation (c.195T >G, p.Y65X) in affected individual (IV:12) (**Figure 1E**). It is predicted that discoidin domain of the retinoschisin would be deleted due to the nonsense mutation. Because we found that three of seven uncles had significant similarities in their clinical manifestations, direct DNA sequencing analysis confirmed the same mutation. Therefore, c.195T >G mutation in *RS1* is responsible for the affected members in this family.

### Generation of *RS1-*KI Mice Models

**FIGURE 2:**
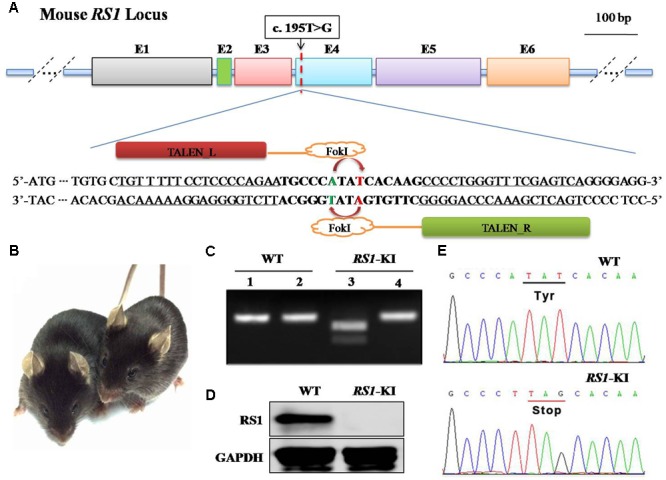
Generation of *RS1*-KI mice. **(A)** Schematic diagram showing the strategy to generate *RS1* knock-in mice. The *RS1* gene has the mutation identified in the proband. We introduced a silence mutation at 192, thus we could use Bpu10I (5′-CCTNAGC-3′, cutting site) to screening the mice. **(B)** *RS1*-KI mice did not show notable physical abnormalities except the eye disease. Right, WT (wide type) mice; left, *RS1*-KI. **(C)** Genotyping results with restriction enzyme. Lines 1 and 2 represent WT, Lines 3 and 4 represent the *RS1*-KI male mice. Lines 1 and 3 represent PCR products digested with Bpu10I. **(D)** The expression of *RS1* in retina of WT and *RS1*-KI mice. **(E)** The results of DNA sequencing.

To recapitulate disease in this XLRS family, we introduced DNA double-strand breaks at the corresponding location of mouse genome via TALEN. Meanwhile, we provided synthetic oligonucleotides harboring the mutation as a donor DNA for homologous recombination (or homology directed repair, HDR). Specifically, a pair of TALEN was designed to target the 4th exon of the mouse *RS1* gene is shown in **Figure 2A**. To quick screen the knock-in mice, we added a Bpu10I site into the donor DNA (**Figure 2A**) harboring the silent mutation. *In vitro* transcribed mRNA for each TALEN-pair was injected into the cytoplasm of one-cell stage mouse embryos. After injection, the embryos were transferred into pseudopregnant female mice.

The genomic DNA of the pups was extracted from tail or toe clips (**Figure 2C**), and the fragments harboring the target site was amplified by PCR. The 309-bp PCR products were obtained for genotyping. The purified products were subjected to digestion with Bpu10I (**Figure 2C**). The sequence of amplicon was also obtained via Sanger sequencing (**Supplementary Figure S1A**). These results revealed that the mutation was successfully knock-in at the target site via TALEN and donor DNA. Specifically, we found two types of the mutation. Four mice have the exact mutation found in the patients (**Supplementary Figure S1B**, Tim1 and **Figure 2E**), we named these mice as *RS1-*KI mice. The other dominant type is 2-bp deletions (**Supplementary Figure S1B**, Tim2), which was triggered by TALEN and was one type of the NEHJ products. *RS1-*KI mice were selected for further characterization. We found that the *RS1-*KI mice were viable, fertile and did not show notable physical abnormalities (**Figure 2B**). Not surprisingly, RS1 protein hasn’t been detected in *RS1*-KI mouse retina with RS1-3R10 antibodies (**Figure 2D**).

### Characterization of *RS1*-KI Mice

**FIGURE 3:**
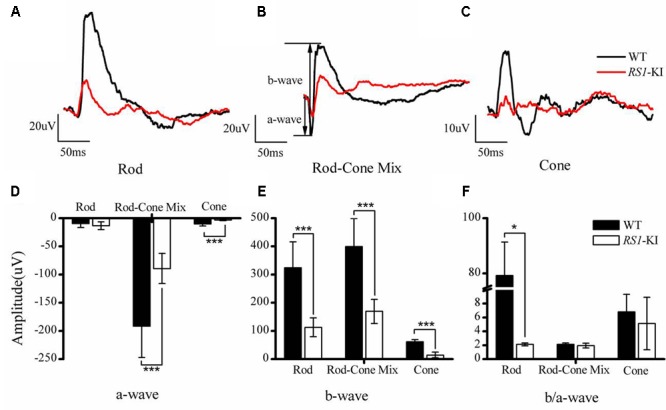
Electroretinography recordings from *RS1*-KI mice. **(A)** The ERG results of rod photoreceptors in WT (black line) mice and *RS1*-KI mice (red line). Compared with WT, the ERG b-wave amplitude in the *RS1*-KI mice is notably reduced. **(B)** The ERG test of Rod-Cone mix, both a-wave and b-wave reduced. **(C)** Cone function of WT mice and *RS1*-KI mice. Compared with WT, the ERG b-wave amplitude of the *RS1*-KI mice nearly disappeared. Statistical analysis of the ERG a-wave amplitude **(D)**, b-wave amplitude **(E)** and b/a ratio **(F)** between WT and *RS1*-KI mice. ^∗^*P* < 0.05; ^∗∗∗^*P* < 0.001.

To assess the physiologic characteristics of *RS1-*KI mice, we performed a series of examinations, including ERG, OCT and retinal morphology. ERG, an objective assessment of retinal function, showed an abnormal response in *RS1-*KI mice at P40. The b wave of ERG dramatically reduced in all dark and light adapted tests (**Figures 3A–C**, red line) and a-wave also reduced. The b-wave but not a-wave amplitude showed significantly reduction in all test (*P* < 0.001, *U*-test, **Figures 3D,E**). The b/a ratio analysis of rod (*P* < 0.05, *U*-test, **Figure 3F**) shows significant differences between the wild type mice and the *RS1*-KI mice. It revealed that the signal of the vision has been blocked from out plexiform layer to inner nuclear layer. These results demonstrated that *RS1*-KI mice have retina dysfunction and the disruption have been occurred in synaptic connections between photoreceptors and bipolar cells.

**FIGURE 4:**
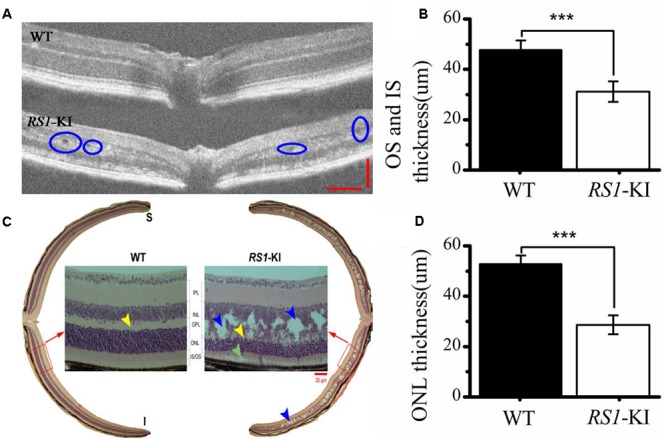
Histological staining and OCT analyze of retina. **(A)** Cavities are observed (blue circle) in the retina of *RS1*-KI mice at 4 weeks compared with WT mice. **(B)** Statistical analysis shows the thickness of OS and IS became significantly shorted in *RS1*-KI mice, compared with WT. **(C)** Retina section exhibits structural changes that include mislocalization (cyan arrows) and dissection through the inner nuclear layer (blue arrows), poorly formed and irregular outer plexiform layer (yellow arrow in *RS1*-KI).OS, outer segment of photoreceptor; IS, inner segment of photoreceptor; ONL, outer nuclei layer; OPL, outer plexiform layer; INL, inner nuclear layer; IPL, inner plexiform layer; RGC, retinal ganglion cell. **(D)** The outer nuclear layer of *RS1*-KI mice is much thinner than that of WT. ^∗∗∗^*P* < 0.001.

Furthermore, we used OCT (**Figure 4A**), hematoxylin and eosin (H&E) staining (**Figure 4C**) to investigate the retinal structure of the *RS1*-KI mice. *In vivo* images of *RS1*-KI mice with OCT showed cavities in the outer plexiform layer (blue circle, **Figure 4A**) at the age of 4 weeks. The IS and OS of photoreceptors became indistinct, which led to the outer retina reflective bands disappeared at cyan arrow zone (**Supplementary Figure S2**). The results of H&E staining indicated a serious split in outer plexiform layer and inner nuclear layer of *RS1*-KI mice (the blue arrow pointing, **Figure 4C**) at the age of 6 weeks. The structure of inner nuclear layer and outer plexiform layer is disorganized, large cavities were observed at the inner nuclear layer. We also observed the apoptosis of the outer segments and the shrink of inner segments, compared with control mice. Notably, some clumps of photoreceptor nuclei were migrated into the inner and outer segments zones. Outer limiting membrane was still visible, albeit overall thickness of ONL was slightly decreased in the *RS1*-KI mice. We also found a trend of reduced thickness of the photoreceptor outer segments and inner segments (*P* < 0.001, *U*-test, **Figure 4B**), the thickness of outer nuclear layer also decreased in the *RS1*-KI mice retina (*P* < 0.001, *U*-test, **Figure 4D**).

**FIGURE 5:**
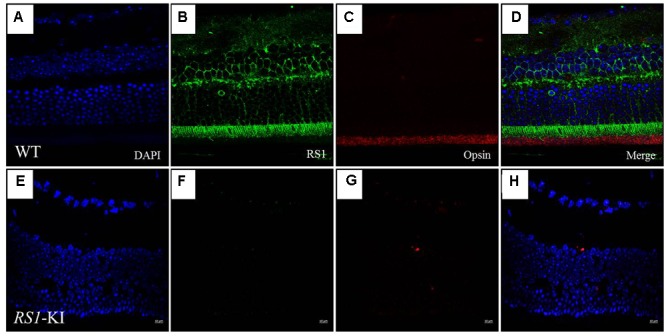
Immunofluorescence imaging of retina from *RS1*-KI mice at 18 weeks. **(A)** All layer labeling with DAPI in WT. **(B)** Retina stained with the RS1 antibody (green). Inner segments of photoreceptors (IS), outer nuclear layer (ONL), outer plexiform layer (OPL) and inner nuclei layer (INL). **(C)** The outer segments of the photoreceptor (OS) stained with the Opsin antibody (red). **(D)** Image is merged with DAPI, RS1 and Opsin. **(E–H)** Immunofluorescence signal was evaluated in the *RS1*-KI mice at 18 weeks. Scale bar, 10 μm.

With the staining with RS1 (Sigma, Germany, 1:100) and Opsin (Sigma, Germany, 1:200) antibodies, we observed the co-location of two proteins in the segments of photoreceptors (**Figure 5**) at age of 18 weeks. Photoreceptor inner segments and bipolar cells have high *RS1* expression level (**Figures 5B,D**), the outer plexiform layer has relative lower expression of *RS1*. More cells have been observed at RGC layer, compared to the WT. Surprisingly, we found that the retinal structure of the *RS1*-KI mice has disordered, including the disarray of outer nuclear layer, chaos of outer plexiform layer, decreased inner segments and loss of outer segments. Taken together, these results showed a novel nonsense mutation (c.195T >G, p.Y65X) is the causative mutation; *RS1*-KI mice have the dysfunctions and abnormal structure of retina, which is similar as clinical manifestations of the XLRS patients.

## Discussion

Genome editing technology holds immense promises for bringing gene therapy into the clinics. At present, there are three XLRS mouse models targeting the murine ortholog of the human *RS1* gene, which have been developed at present (**Supplementary Table S1**). All three knock out models display similar features (Weber et al., 2002; Zeng et al., 2004; Jablonski et al., 2005), with hallmarks of the human disease, but the special mutations from the models have not been identified in patients. These knock-out mice via insertion of exogenous antibiotics gene or large deletion leads to the production of truncated RS1 protein or loss of RS1 protein. These models could be harnessed for the development of traditional gene therapy via ectopic expression of the wild-type *RS1* gene. While, there are many problems in traditional gene replacement therapy, such as gene silence and random integration. With the development of biotechnology, genome editing based gene therapy has obvious advantage; it corrects *in situ*, thus it solves the gene silence and random integration issue. To develop genome editing based gene therapy, knock-in animal harboring the identical mutation found in the patients is critical, and that these methods which developed in the knock-in mice could be easily transferred to the patients.

To date, Zinc-finger nucleases (ZFNs), TALENs, and Clustered Regularly Interspaced Short Palindromic Repeats (CRISPRs)/Cas9 have greatly facilitated the generation of animal disease models. Here, we used TALEN to knock-in the mutation (c.195T >G, p.Y65X) in fertilized mouse eggs. Unlike ZFN, which is only able to recognize sequences ranging from 9 to 18 nucleotides (Maeder and Gersbach, 2016), TALEN has overcame the specific sequences limitation. Thus, we used TALEN to introduce DNA double-strand breaks in the mouse *RS1* locus and the donor DNA harboring the mutation and restriction enzyme site as repair template. Our original plan is to screen the mice conveniently in the further experiments with the additional restriction enzyme site. Actually, it did not require such design of TALENs, because highly efficient TALENs could allow us to get enough desired mutant founders. While, we found that the most pups are chimera, which highlights the TALENs related chimera issue should be solved. More recently, CRISPR/Cas9 represents a new genome editing technology for generation of the animal models for human disease. Off-target is still a key bottleneck, which leading to modifications of the genome at undesired locations (Barrangou and Doudna, 2016). With the development of high-fidelity mutants (Kleinstiver et al., 2016; Kulcsar et al., 2017), CRISPR/Cas9 will serve as another genome editing tool for generation of human disease model. Our recent study shows that FnCpf1 can be harnessed for genome editing in human cells (Tu et al., 2017). Our ongoing project is using FnCpf1 to correct the present mutation to treat this disease with the knock-in mice disease model.

In this study, we found the proband has a serious vascular attenuation or sheathing, retinal pigment deposition (**Figure 1B**), which are similar to age-related macular degeneration (AMD). XLRS is an inherited developmental retinal disease, which is the leading cause of vision loss from macular degeneration in young men. As previously reported, loss and dysfunction of RS1 triggered disturbances in retinal photoreceptors and bipolar cells. Eventually, the macular alterations may be presented from the characteristic spoke-wheel pattern to unspecific mild retinal pigment abnormalities (Molday et al., 2012). We also observed the degeneration of retinal pigment deposition, a process of gradual pathogenesis. We found, as to the mouse disease model, the RPE didn’t degenerate at the early stages (at 6 weeks), but abnormal retinal morphology were detected, including spitted inner nuclear layer, shorted inner segments and reduced number of outer segments (**Figure 4** and **Supplementary Figure S2**). Striking morphological abnormalities were observed at 18 weeks (**Figures 5E–H**). It revealed that dysfunction of the retinoschisin may result in the apoptosis of RPE cells and outer segments of photoreceptors. These results demonstrated that the photoreceptor retrograded apoptosis may lead to the macular degeneration exhibiting progressive degeneration in the *RS1*-KI mice. So we could treat XLRS as “inner-retinal initiation” disease. AMD (age-related macular degeneration), is a leading cause of vision loss in the United States and it destroys the macula. In the early stages of AMD, which is asymptomatic, insoluble extracellular aggregates called drusen accumulate in the gap of RPE cells (Ambati and Fowler, 2012). Thus, AMD represents “outer-retinal initiation” macular degeneration. The different initiation of the retinal pigment deposition will be helpful for us to understand the molecular insights of XLRS and AMD.

In summary, our studies demonstrated that a novel nonsense mutation in *RS1* gene (c.195T >G) would lead to human retinoschisis. We also generated *RS1*-KI mouse model, which would be very useful for genome editing based gene therapy as well as elucidation of molecular mechanism.

## Ethics Statement

This study conformed to the tenets of the Declaration of Helsinki. It was approved by the Ethics Committee of Eye Hospital, Wenzhou Medical University. Written informed consent was obtained from the recruited individuals. All experiments were performed in accordance with the approved guidelines. This study was performed in strict accordance with the recommendations in the Guideline for the Care and Use of Laboratory Animals from the National Institute of Health. The protocols were specifically approved by the Committee on the Ethics of Animal Experiments of the Wenzhou Medical University.

## Author Contributions

FG and JZ designed the experiment. DC, MT, and TX performed the experiments. DC, JX, RY, TY, WZ, and JL provided the clinical evaluation. TX, MT, CZ, XH, RD, XG, ZS, and JZ analyzed the data. FG and TX wrote the paper. All authors were involved in revising the paper for important intellectual content, and gave final approval of the version to be published.

## Conflict of Interest Statement

The authors declare that the research was conducted in the absence of any commercial or financial relationships that could be construed as a potential conflict of interest.

## Footnotes

1 http://www.dmd.nl/rs/index.html

2 http://sift.jcvi.org/

3 http://genetics.bwh.harvard.edu/pph2/

4 http://www.pantherdb.org/tools/csnpScoreForm.jsp

5 http://espript.ibcp.fr/ESPript/cgi-bin/ESPript.cgi

## Supplementary Material

FIGURE S1 Results of sequences alignment. **(A)** Sanger sequencing analysis of different genotypes. Two amino acids were deleted in Mouse-18 and Mouse-22. *RS1* gene has a mutation site at c.195 T >G in Mouse-26, Mouse-27, Mouse-28, Mouse-30. WT, wild type; M, DNA marker. **(B)** The two types of mutation. TM1 is the *RS1*-KI mice. The other type is 2-bp deletions, which was triggered by TALEN and was one of the NEHJ products in TIM2.

FIGURE S2 Retinal structure in OCT images of *RS1*-KI mice. **(A)** OCT images show four outer retina reflective bands distal to the ONL, labeled 1, 2, 3, and 4. These bands align with structures in the photoreceptor and RPE. **(B)** OCT image from an age-matched (4 weeks) *RS1*-KI mice with multiple cavities (blue arrow) spanning the INL and OPL, making the border between OPL and INL and between OPL and ONL uneven and less clear. The ONL is also decreased in thickness. Moreover, the second and third outer retina bands in the OCT images are replaced by a single wider reflective band (cyan arrow). Scale bars, 50 μm.

TABLE S1 Comparison of the XLRS mouse models.
