# Supplementary figures and images for: Recapitulating X-Linked Juvenile Retinoschisis in Mouse Model by Knock-In Patient-Specific Novel Mutation
Source: Front Mol Neurosci. 2018 Jan 12;10:453. doi: 10.3389/fnmol.2017.00453 (PMC5770790; doi:10.3389/fnmol.2017.00453)

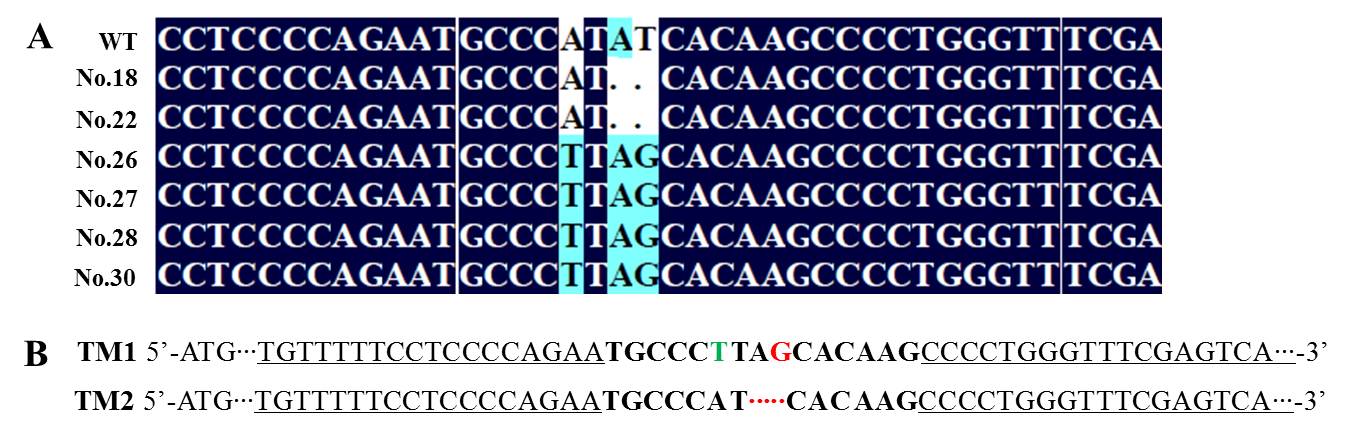

Supplement: FIGURE S1 — Results of sequences alignment. (A) Sanger sequencing analysis of different genotypes. Two amino acids were deleted in Mouse-18 and Mouse-22. RS1 gene has a mutation site at c.195 T >G in Mouse-26, Mouse-27, Mouse-28, Mouse-30. WT, wild type; M, DNA marker. (B) The two types of mutation. TM1 is the RS1-KI mice. The other type is 2-bp deletions, which was triggered by TALEN and was one of the NEHJ products in TIM2. [file Image_1.JPEG]

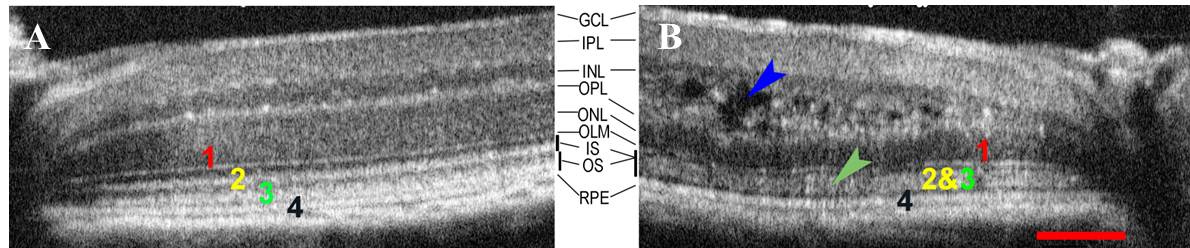

Supplement: FIGURE S2 — Retinal structure in OCT images of RS1-KI mice. (A) OCT images show four outer retina reflective bands distal to the ONL, labeled 1, 2, 3, and 4. These bands align with structures in the photoreceptor and RPE. (B) OCT image from an age-matched (4 weeks) RS1-KI mice with multiple cavities (blue arrow) spanning the INL and OPL, making the border between OPL and INL and between OPL and ONL uneven and less clear. The ONL is also decreased in thickness. Moreover, the second and third outer retina bands in the OCT images are replaced by a single wider reflective band (cyan arrow). Scale bars, 50 μm. [file Image_2.JPEG]
