# Supplementary material for: Recapitulating X-Linked Juvenile Retinoschisis in Mouse Model by Knock-In Patient-Specific Novel Mutation
Source: Front Mol Neurosci. 2018 Jan 12;10:453. doi: 10.3389/fnmol.2017.00453 (PMC5770790; doi:10.3389/fnmol.2017.00453)
Supplement: TABLE S1 — Comparison of the XLRS mouse models. [file Table_1.DOC]

**Supplement Table 1 Comparison of the XLRS mouse models**

| **Name** | **Source** | **Target** | **Category** | **Disease phenotypes** | **Organization** |
| --- | --- | --- | --- | --- | --- |
| *Rs1h* ^-/Y^ | Weber B.F, *et al*. 2002 | Partial deletions of exon 3 and 4 and intron 3 with a *Lac Z-Neo^r^* gene insertion, a null mouse model | Targeted (knock out); C57BL/6 (murine ortholog of *RS1* gene) | Splitting of the INL and ONL; overall disorganization of retinal cell layers with irregular displacement of cells; loss b-waves; loss of the synaptic in the IPL | Nervures system, vision/eye |
| *Rs1*^-/Y^ and *Rs1*^-/-^ | Zeng Y., *et al*. 2004 | Deletion of all of exon 1 and partial deletion of intron 1 with a neomycin gene insertion | Targeted (knock out); C57BL/6 (murine ortholog of *RS1* gene) | Retinoschisis cavities in the INL; b-wave reduction; disorganization of multiple retinal layers | Vision/eye |
| *44TNJ Rs1*^tmgc1^/*Rs1^t^*^mgc1^ and *Rs1*^tmgc^/Y formerly called *Rs1h*^tmgc1^ | Jablonski M.M., *et al*. 2005 | A 10 bp insertion at the first base with a T>C exchange at the second base of intron 2; a 26 bp deletion at the junction of exon 2 to intron 2 of the *Rs1h* gene | Chemically induce (*N*-ethyl-*N*-nitrosurea, ENU); Tennesse mouse genome consortium | Abnormal fundus phenotype (intraretinal microflecks); disruption of the lamination in retina; focal areas of inner nuclear layer splitting | Nervures system, pigmentation, eye |
| *RS1*-KI | In present study | A T>G mutation at the 195 amino acid of exon 4 of the *RS1* gene; missense mutation | Targeted (knock in); C57BL/6J (humanized mutation site at the murine ortholog of *RS1* gene) | Cavities in the OPL and INL; ONL, OS+IS thicknesses; overall disorganization of retinal cell layers; b wave of ERG disappeared | Vision/eye |
